# Toll-Like Receptor Agonists Synergize with CD40L to Induce Either Proliferation or Plasma Cell Differentiation of Mouse B Cells

**DOI:** 10.1371/journal.pone.0025542

**Published:** 2011-10-03

**Authors:** Emmanuelle Boeglin, Cristian R. Smulski, Susana Brun, Sara Milosevic, Pascal Schneider, Sylvie Fournel

**Affiliations:** 1 CNRS UPR 9021, Immunologie et Chimie Thérapeutiques, Institut de Biologie Moléculaire et Cellulaire (IBMC), 67084, Strasbourg, France; 2 Department of Biochemistry, University of Lausanne, Ch. des Boveresses 155, Epalinges, Switzerland; University of London- St George's, United Kingdom

## Abstract

In a classical dogma, pathogens are sensed (via recognition of Pathogen Associated Molecular Patterns (PAMPs)) by innate immune cells that in turn activate adaptive immune cells. However, recent data showed that TLRs (Toll Like Receptors), the most characterized class of Pattern Recognition Receptors, are also expressed by adaptive immune B cells. B cells play an important role in protective immunity essentially by differentiating into antibody-secreting cells (ASC). This differentiation requires at least two signals: the recognition of an antigen by the B cell specific receptor (BCR) and a T cell co-stimulatory signal provided mainly by CD154/CD40L acting on CD40. In order to better understand interactions of innate and adaptive B cell stimulatory signals, we evaluated the outcome of combinations of TLRs, BCR and/or CD40 stimulation. For this purpose, mouse spleen B cells were activated with synthetic TLR agonists, recombinant mouse CD40L and agonist anti-BCR antibodies. As expected, TLR agonists induced mouse B cell proliferation and activation or differentiation into ASC. Interestingly, addition of CD40 signal to TLR agonists stimulated either B cell proliferation and activation (TLR3, TLR4, and TLR9) or differentiation into ASC (TLR1/2, TLR2/6, TLR4 and TLR7). Addition of a BCR signal to CD40L and either TLR3 or TLR9 agonists did not induce differentiation into ASC, which could be interpreted as an entrance into the memory pathway. In conclusion, our results suggest that PAMPs synergize with signals from adaptive immunity to regulate B lymphocyte fate during humoral immune response.

## Introduction

Immune response activation by pathogens involves various receptors, known as pattern recognition receptors (PRRs) that recognize pathogen-specific molecular patterns (PAMP for “Pathogen Associated Molecular Pattern”) (reviewed in [1]). Among the seven classes of PRRs, the best characterized class is Toll-Like Receptors (TLRs), a family of type I transmembrane proteins characterized by highly divergent leucine-rich extracellular domains and highly conserved Toll-IL-1R cytoplasmic domains. To date, 11 mouse and human TLRs have been described allowing detection of specific pathogen markers that included lipopolysaccharide (LPS), double-standed RNA, peptidoglycan and hypomethylated DNA (reviewed in [2]). Most TLRs (TLR 1, 2, 4, 5, 6, 10 and 11) are expressed on the cell surface, whereas others TLRs (TLR 3, 7, 8 and 9) are present in endosomal compartments. Furthermore, TLRs are expressed as homodimers or as heterodimers (TLR1+TLR2 or TLR2+TLR6). Finally, TLRs are linked by adapter molecules (MyD88 or TRIF) to intracellular signaling pathways that generally lead to transcription of NF-kB and IRF target genes.

Pathogen recognition by PRRs activates innate immune cell, including dendritic cells that can in turn activate the adaptive immune response. Moreover, many reports described that TLR expression on lymphocytes allowed direct activation of adaptive immune responses by PAMPs, such as B cell activation and differentiation into antibody secreting cells (ASC). It was described years ago that activation of the TLR4 pathway by LPS triggers mouse B cell proliferation, differentiation into ASC and isotype switch [3]. More recently, using mice deficient for MyD88, Pasare *et al.* [4] showed that direct TLR-signal is essential for B cell activation and differentiation into ASC. These results were opposed by Gavin *et al.* [5] who showed, by using mice deficient in both MyD88 and TRIF, that direct-TLR signal was not necessary for B cell activation and differentiation. Finally, Meyer-Bahlburg *et al.* [6] showed that B-cell intrinsic TLR signals amplified but were not required for humoral immunity. In the same way, various *in vitro* assays using mouse B cells showed that TLR1/2 or 2/6 [7]–[11], TLR3 [12], TLR4 [7]–[8], [10], TLR7 [13]–[14], TLR8 [15] and TLR9 [7]–[8], [10] agonists induced B cell proliferation, expression of activation markers and cytokine production while TLR2 [7]–[10], TLR4 [8], [10], TLR7 [8], [10] and TLR9 [8], [10] agonists induced B cell differentiation into ASC. Of note, TLR5 expression on mouse B cells is still controversial and B cell activation or differentiation by TLR5 agonists has never been described [8], [10]. Similarly, various reports have led to the conclusion that mouse TLR8 is nonfunctional [16]–[17]. More recently, TLR8 has been described as a negative regulator of TLR7-induced immune response [18].

Beside these newly described innate stimuli, the current dogma postulates that 2 signals are necessary to drive T-dependent naive B cell proliferation and differentiation into ASC: recognition of an antigen by the B cell-specific receptor (BCR) and a T cell co-stimulatory signal in which CD40L (CD154) expressed on activated T cells activates CD40 on B cells. Interaction of CD40 with CD154 is essential for antibody production and isotype switch, and therefore for an optimal humoral immune response. Moreover, various studies showed that dual stimulation of B cells through BCR and CD40 leads to an enhancement of antibody and cytokine production (reviewed in [19]–[20]). However, the effects and mechanisms of interactions between innate immune receptors and BCR or CD40 are not yet well defined. Some reports showed that addition of CD40 signal to TLR7 or TLR9 agonists enhanced cytokine production [21]–[22] as well as B cell differentiation into ASC [22]–[23] in both human and mouse primary B cells. These effects were increased after addition of a BCR signal. In contrast, Knödel et al. [24] showed that although TLR4 agonists synergize with CD40L to induce plasma cell differentiation, addition of BCR signal results in suppression of B cell differentiation into ASC.

B cells play an important role in protective immunity [25] as producers of both antibodies and cytokines. Activation of naive B cells results in expression of activation marker (such as CD69 and CD86), production of cytokines (for example CCL22 [26]) and proliferation. Among proliferating cells, some differentiate into ASC and others into memory B cells. Some cells migrate to germinal centers to finish their differentiation and produce highly efficient neutralizing antibodies. To this end, the affinity maturation of antibody response is required and involves immunoglobulin class switch recombination (CSR) that directs antibody production from IgM to IgG, IgA and IgE and somatic hypermutation (SHM) that modifies the Ig variable region gene to obtain BCR with high affinity for antigen. Both events are controlled by the activation-induced cytidine deaminase (AID) (Reviewed in [27]). CD138 (Syndecan-1), a proteoglycan that recognizes extracellular matrix and growth factors, is present on ASC and is often used as marker of plasma cells [28]. However, as CD138 is also expressed on plasmablasts [29] prior to the fully differentiated plasma cells, ASC are characterized not only by a high expression of CD138 but also by a low expression of B220 molecule [30]. Differentiation to ASC correlates with the expression of a number of transcription factors among which B lymphocyte-induced maturation protein 1 (Blimp-1) has been proposed to be the master regulator [31]. Conversely, the transcription factor Pax5 is associated with the mature B cell phenotype and downregulated in ASC [32].

Here, we systematically screened the effects of combined activation of TLRs, CD40 or the BCR on B cell proliferation, activation and differentiation into ASC. To this end, mouse spleen B cells were activated by different TLR agonists alone or in the presence of recombinant mouse CD40L and/or anti-BCR antibodies to mimic antigen. As expected, mouse B cells stimulated with TLR agonists were activated, proliferated and differentiated into ASC. Nevertheless, differentiation to ASC was not observed for TLR3 and TLR9 agonists. Addition of a CD40 signal to TLR-stimulated B cells induced either increased B cell proliferation and activation (TLR3, TLR4, and TLR9) or increased B cell differentiation into ASC (TLR1/2, TLR2/6, TLR4 and TLR7). Furthermore, differentiation into ASC was not increased by addition of BCR signal after stimulation of CD40 and TLR3 or TLR9.

Taken together, our results suggest that signals originating directly from pathogens interact with signals from adaptive immunity to regulate B lymphocyte fate during humoral immune responses.

## Results

### TLR-3, -4 and -9 but not TLR -1/2, -2/6 and -7 agonists synergize with CD40L to increase mouse spleen B cell proliferation and activation

To evaluate the effect of dual B cell stimulation by both innate and adaptive immune signals on cell proliferation/survival and activation, mouse spleen B cells were cultured in the presence of increasing concentration of TLR and CD40 agonists. [^3^H]thymidine incorporation, cell surface expression of CD69 and CD86, and CCL22 production (the first cytokine produced during B cell activation [26]) were measured. TLR2/6 pathway was activated by Pam2CSK4, TLR1/2 by Pam3CSK4, TLR3 by poly (I:C), TLR4 by lipopolysaccharide (LPS), TLR7 by R848 and TLR9 by unmethymated CpG oligodeoxynucleotides type C (ODN 2395 described to activate B and dendritic cells [33]). CD40 pathway was activated by recombinant mouse CD40L (mCD40L).

**Figure 1:**
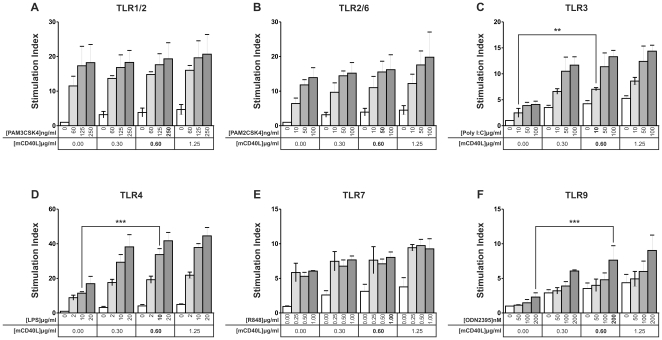
Proliferative response of mouse splenic B cells induced by TLR agonists in association with mCD40L. Purified B cells were stimulated with TLR1/2 agonist (Pam3CSK4) (A), TLR2/6 agonist (Pam2CSK4) (B), TLR3 agonist (Poly (I:C)) (C), TLR4 agonist (LPS) (D), TLR7 agonist (R848) (E), TLR9 type C agonist (ODN 2395) (F) at the indicated concentrations and in association with increasing concentrations of mCD40L for 72 h. Proliferation was evaluated by measuring [^3^H]-thymidine uptake. Data are expressed as mean Stimulation Index (see material and methods) ± SD of three independent experiments. The mean of cpm in non activated B cells is 309±34. For C, D and F, although addition of mCD40L at all the concentrations induced significant changes, statistics are only shown for the concentrations used in further experiments (bold letters). *p<0.05, **p<0.01, ***p<0.001.

As expected, our data indicated that, in the absence of mCD40L, agonists of TLR 1/2, 2/6, 4 and 7 but not agonists of TLR3 and TLR9 induced strong mouse B cell proliferation (**Figure 1**). Although mCD40L alone induced only a slight proliferative response (**Figure 1** **, white bars**), addition of mCD40L to TLR3 (**Figure 1C**), TLR4 (**Figure 1D**) and TLR9 (**Figure 1F**) agonists induced an increase in mouse B cell proliferative response. In contrast, mCD40L addition had no significant effect on TLR1/2- (**Figure 1A**), - TLR2/6 (**Figure 1B**) and TLR7- (**Figure 1E**) induced B cell proliferative response. The specificity of mCD40L action was confirmed in CD40-deficient splenic B cells (**Figure S1A**). Moreover, Polymixin B, an inhibitor of LPS activity inhibited LPS-induced proliferative response with or without mCD40L addition but had no effect on TLR3-induced proliferation as well as on the increase of this proliferative response in the presence of mCD40L (**Figure S1B**), showing the specificity of each TLR agonist and ruling out the possibility of a mCD40L-independent LPS-dependent B cell proliferation.

The proliferative response measured by thymidine uptake quantifies not only the activity of dividing cells but also the survival rate of proliferating cells. CD40L is known to promote both proliferation and survival, although we didn't attempt to distinguish both effects in this study. In CFSE labeling experiments, an increase in cell survival as well as in the numbers of dividing cells was observed in the conditions in which an increase of thymidine uptake was observed (Data not shown).

**Figure 2:**
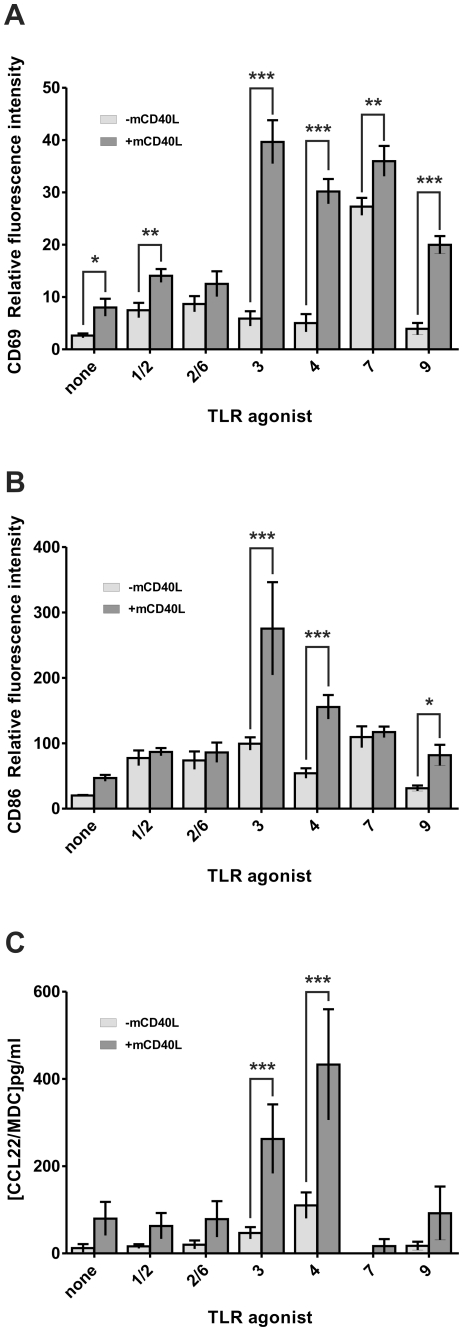
B cell activation induced by TLR agonists in association with mCD40L. Purified B cells were cultured with TLR1/2 agonist Pam3CSK4 (250 ng/mL), TLR2/6 agonist Pam2CSK4 (50 ng/mL), TLR3 agonist poly (I:C) (10 µg/mL), TLR4 agonist LPS (10 µg/mL), TLR7 agonist R848 (1 µg/mL) or TLR9 agonist ODN 2395 (200 nM) alone (light gray bars) or in the presence of 0.6 µg/mL mCD40L (dark gray bars). After 24 h, expression of activation markers CD69 (A) or CD86 (B) were measured by flow cytometry. Results are expressed as mean relative fluorescence intensity. Mean values ± SD of three independent experiments are shown. (C) CCL22/MDC levels were measured in cell culture supernatants by ELISA after 48 h. Results are expressed in pg/mL and represent means ± SD of three independent experiments. *p<0.05, **p<0.01, ***p<0.001.

B cell activation induced by TLR agonists and mCD40L was evaluated by monitoring CD69 and CD86 surface expression as well as CCL22 production. As expected, all TLR agonists tested taken alone increased activation marker expression (CD69; **Figure 2A** or CD86; **Figure 2B**). Only a fraction of B cells increased CD69 expression (from 10% in non treated cells to 85% in fully activated cells) whereas all B cells increased CD86 expression. Production of CCL22, was increased after activation by TLR3 and TLR4 agonists alone (**Figure 2C**). As seen for the proliferative response, co-treatment with mCD40L increased CD69 (**Figure 2A**) and CD86 (**Figure 2B**) expression as well as in CCL22 production (**Figure 2C**) in response to TLR3 and TLR4 agonists and to a lower extent to TLR9 agonist, but no or little to TLR1/2, TLR2/6 and TLR7 agonists. As for proliferative response, mCD40L alone only slightly increased activation marker expression and CCL22 production (**Figure 2**).

In conclusion, our data demonstrate that the CD40 pathway synergizes with TLR3, 4 and 9 pathways, but not with TLR1, 2, 6 and 7 for B cell proliferative response and activation (**Table 1**).

**Table 1:** Summary of B cell activation and/or differentiation induced by TLR agonist in association with mCD40L.

		None	1/2	2/6	3	4	7	9
Total B cells	Prolif	Ns	ns	ns	++	+++	ns	+++
	CD69	+	++	ns	+++	+++	++	+++
	CD86	Ns	ns	ns	+++	+++	ns	+
	CCL22	Ns	ns	ns	+++	+++	ns	ns
Total	BLIMP-1	Ns	+	++	ns	+	+	ns
Enriched CD138+	CD138^high^ B220^low^	Ns	+++	+++	ns	ns	+++	ns
	AID	Ns	+	++	ns	ns	+	ns
Total B cells	IgM	++	+++	ns	+	ns	+++	+++
	IgG	Ns	+++	ns	ns	+++	ns	+

Results represent the difference between the treatment with TLR agonists alone in comparison with the co-treatment TLR agonists plus mCD40L. P<0.001 +++, P<0.01 ++, P<0.05 +, P>0.05 ns.

### TLR -1/2, -2/6, - 4 and -7 but not TLR-3 and -9 agonists synergize with CD40L to increase mouse spleen B cell differentiation in plasma cells

We next examined the potential synergy of TLRs and CD40 pathways on antibody production, B cell differentiation into ASC and affinity maturation of antibody response.

**Figure 3:**
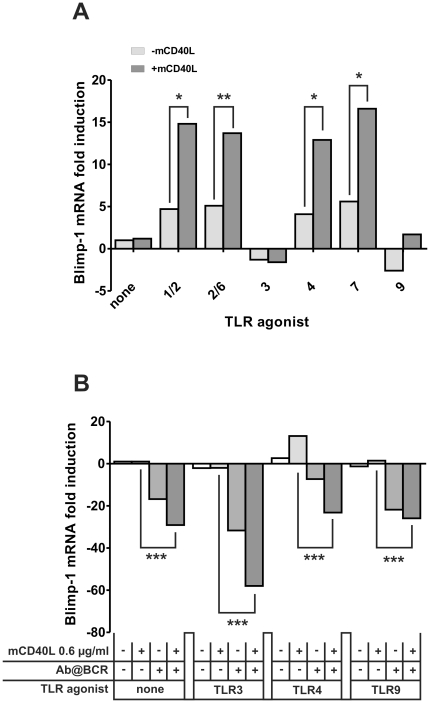
Blimp-1 mRNA expression in splenic B cells induced by TLR agonists in association with mCD40L. Purified B cells were cultured with the indicated TLR agonists (at the concentrations of figure 2) alone (light gray bars) or in the presence of 0.6 µg/mL of mCD40L (dark gray bar) (A) or with 10 µg/mL of TLR3 agonist poly (I:C), 10 µg/mL of TLR4 agonist LPS or 200 nM of TLR9 agonist ODN 2395 with or without 0.6 µg/mL of mCD40L and 1 µg/mL anti-IgM antibody, as indicated (B). After 72 h, total RNA was isolated and expression of Blimp-1 transcripts was evaluated by quantitative real-time PCR. Values were normalized to the mean of expression of three housekeeping genes: GAPDH, β-actin, HPRT. Results are expressed as the fold induction of gene transcription as compared to purified B cells cultured in medium. Data of one representative experiment out of three is shown. *p<0.05, **p<0.01, ***p<0.001.

Mouse spleen B cells were co-stimulated with TLR agonists and mCD40L. Differentiation into ACS was monitored by following mRNA expression of Blimp-1, the master regulator of ASC differentiation [31]. As shown in **figure 3A**, TLR1/2, TLR2/6, TLR4 and TLR7 agonists induced an increase of Blimp-1 mRNA expression that is enhanced by addition of mCD40L, indicating the triggering of the gene programme for ASC differentiation. Incubation of splenic B cells with mCD40L alone (**Figure 3A**) did not increase Blimp-1 mRNA expression. The specificity of mCD40L action was confirmed in CD40-deficient B cells (**Figure S2A**). Similarly, Blimp-1 mRNA expression induced by TLR2 and TLR4 agonist was inhibited by an antagonist anti-TLR2 antibody (**Figure S2B**) and by polymixin B (**Figure S2C**) respectively, showing that these TLR pathways are implied in Blimp-1 mRNA expression induction.

As shown in **Figure 3A**, TLR3 agonist as well as TLR9 Type C agonist (ODN 2395), described to activate B and dendritic cells [33] or TLR9 Type B agonist (ODN 1668, **Figure S2D**), described to activate B cells [34] did not induce any increase in Blimp1 mRNA expression. Addition of mCD40L to these agonists did not modify Blimp-1 mRNA expression. As it was proposed that full-blown naive human B cell activation requires three synergically acting stimuli, TLR engagement, interaction of CD40 with CD40L and recognition of antigen by the B cell specific receptor (BCR) [35]–[36], an anti-IgM antibody was added to engage the BCR in the presence of TLR3 and TLR9 agonists and mCD40L. As shown in **figure 3B**, the addition of a BCR signal did not increase Blimp-1 expression in B cell cultures stimulated with combinations of mCD40L with TLR3 and TLR9 agonists (**Figure 3B**). On the contrary, stimulation with anti-IgM always decreased Blimp-1 mRNA expression, even with CD40L – TLR4 combinations that induced Blimp-1 when applied alone.

**Figure 4:**
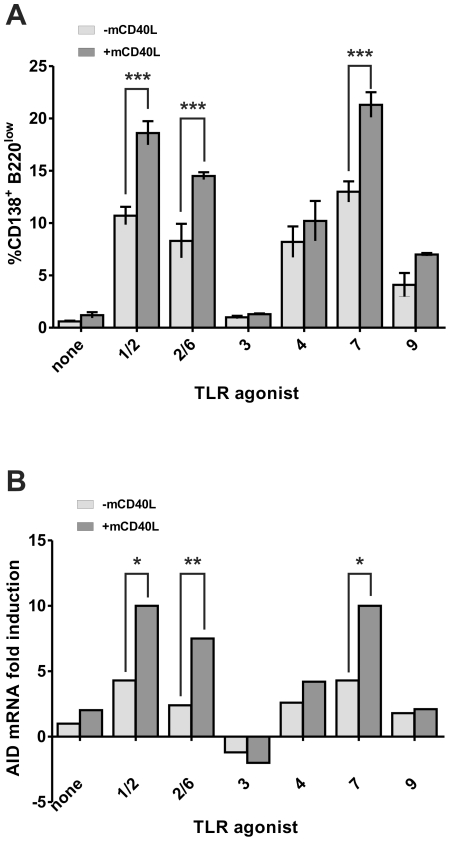
B220 expression and AID mRNA expression in CD138-enriched purified B cell population after activation by TLR agonists in association with mCD40L. Purified B cells were cultured with the indicated TLR agonists (at the concentrations of figure 2) alone (light gray bars) or in the presence of 0.6 µg/mL mCD40L (dark gray bar). After 72 h, the population was enriched for CD138 positive cells as described in *Material and Methods*. (A) CD138-enriched purified B cells were then co-stained with anti-B220 and anti-CD138 antibodies and the expression of these markers was determined by flow cytometry. Results are expressed as % of CD138^+^ B220^low^ +/−SD of three independent experiments. (B) Total RNA was isolated and expression of AID transcripts was evaluated by quantitative real-time PCR. Results are expressed as the fold induction of gene transcription as compared to CD138-enriched purified B cells cultured in medium. *p<0.05, **p<0.01, ***p<0.001.

To characterize more precisely the B cell subset expressing Blimp-1 mRNA, mouse spleen B cells were cultured as described above and then enriched for CD138^+^ cells. These cells were tested for B220 expression by flow cytometry and for Blimp-1, Pax5 and AID mRNA expression by qRT-PCR. The CD138^+^ B cell population contains all plasma cell subsets, including the ASC that are characterized by low expression of the B220 molecule [30]. To evaluate the number of ASC, we quantified B220^low^ cells in the CD138^+^ population. As showed in **figure 4A**, all TLR agonists except TLR3 induced a significant increase in CD138^+^B220^low^ cells, corresponding to ASC subpopulation (**Figure S4**). Addition of a CD40 signal further increased the number of ASC after activation of TLR1/2, TLR2/6 and TLR7 pathways. These results were corroborated by an increase of Blimp-1 mRNA expression, characteristic of ASC (**Figure S3A**), and/or a decrease of PAX-5 mRNA characteristic of mature B cells (**Figure S3B**) in the same conditions.

We next investigated how AID, an enzyme required for CSR and SHM, was induced at the mRNA level in CD138^+^ cells. In the absence of mCD40L, AID mRNA levels was not or minimally modified by treatment with TLR2/6, TLR3, TLR4 and TLR9 agonists and only slightly increased by treatment with TLR1/2 and TLR7 agonists (**Figure 4B**). However, mCD40L increased AID mRNA expression in CD138^+^ B cell-enriched population when added in combination with TLR1/2, 2/6, and 7 agonists (**Figure 4B**).

**Figure 5:**
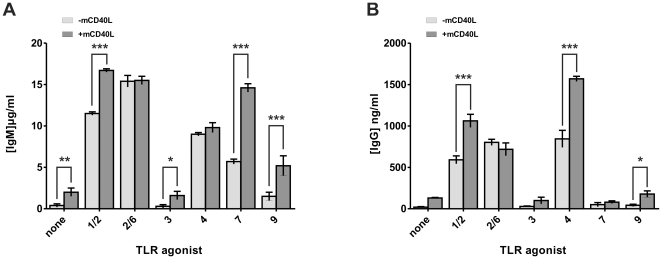
Antibody production in purified B cell culture supernatant after activation by TLR agonists in association with mCD40L. Purified B cells were cultured as described in figure 2 for 6 days. Cell culture supernatants were collected and amounts of IgM (A) and IgG (B) were measured by ELISA as described in *Material and Methods*. Results are expressed in µg/mL for IgM and ng/mL for IgG and shown as the means+SD of three independent experiments. *p<0.05, **p<0.01, ***p<0.001.

Finally, we measured immunoglobulin production in B cell culture supernatants after 6 days of culture with TLR agonists with or without mCD40L. Despite poor B cell survival, IgM and IgG production could be measured in culture supernatants. All TLR agonists alone induced immunoglobulin production except TLR3 and TLR9 agonists for IgM production (**Figure 5A**) and TLR3, TLR7 and TLR9 agonists for IgG production (**Figure 5B**). Addition of mCD40L increased IgM production in B cell culture supernatants after activation by TLR1/2, TLR7 and TLR9 agonists (**Figure 5A**) and IgG production after activation by TLR1/2,TLR4 and to a lower extend TLR9 agonists (**Figure 5B**).

Taken together, these results showed that mCD40L stimulates differentiation into ASC *in vitro*, induces AID mRNA expression and promotes antibody production when combined with TLR1/2, 2/6, 4 and 7 agonists (with the exception of antibody production induced by TLR2/6) (**Table 1**).

## Discussion

According to the classical dogma, full-blown naive B cell activation requires two independent signals: one provided by recognition of antigen by the specific B cell receptor and another provided by T-cell co-stimulation via interaction between CD40L (expressed on activated T cells) and CD40 expressed on B cells. More recently, the finding that B cells express TLRs and can be activated by TLR agonists prompted the modification of this model with a third signal provided directly by pathogens [35] via binding of PAMPs on PRRs. However, the importance of TLR signaling in shaping humoral response remains controversial [4]–[6]. To evaluate the importance of TLRs in B cell response, authors immunized mice that were deficient for the signaling of all (MyD88/TRIF deficient mice [5]) or most (Myd88 deficient mice [4]) TLRs with model antigens in the presence of various adjuvants and evaluated antibodies production. In Myd88 deficient mice, Pasare *et al.* [4] used LPS that binds to TLR4 and have shown a central role for TLR signaling in B cell response. In Myd88/TRIF deficient mice, Gavin *et al.* [5] have used Alum that has been shown later to involve the NOD (Nucleotide binding domain) Like Receptor (NLR) pathway [37]–[38], another family of PRRs, and Freund Complete Adjuvant that activated TLR2 and TLR4 pathway but also the NLR pathway via the Muramyl Dipeptide [39] and have, obviously, shown a poor implication of TLR in antibody production. Moreover, as dendritic cells also express TLRs [2], the effect of TLR deficiency on antibody production can be explained by an indirect effect on DCs. In view of these contradictory results, we tested the direct role of each TLR on B cell activation and antibody production in a systematic *in vitro* assay. Furthermore, as a complete B cell activation requires at least two signals, we dissociated the various signals by activating mouse B cell with TLR agonists alone or associated to the T cell co-stimulation signal CD40L.

As already described [7]–[13], we confirmed that all TLR agonists (except those of TLR5 and TLR8 that are not expressed or not functional or not activating on mouse B cells) induce proliferation and activation of splenic B lymphocytes (**Figure 1** **and** **2**). Surprisingly, the TLR9 agonist type C ODN 2395 described to activate B cell and DC had little effect on B cell proliferation and activation (**Figure 1** **and** **2**). More surprisingly, we found that the TLR3 agonist poly (I:C) as well as the TLR9 agonist type C (ODN 2395) or type B (ODN 1668) did not induce B cell differentiation into ASC and antibody production *in vitro*. Interestingly, addition of a T cell co-stimulation signal via CD40L to TLR agonists shows a dissociation between on the one hand, induction of mouse B cell proliferation and activation and, on the other hand, induction of B cell differentiation into ASC and antibody production (**Table 1**). Indeed, with the exception of the TLR4 agonist LPS that synergizes with mCD40L to increase both proliferative response and differentiation into ASC, all other TLR agonists synergize with mCD40L to increase either a proliferative response associated with activation marker expression and cytokine production (TLR3 and TLR9) or B cell differentiation into ACS and antibody response maturation (TLR 1/2, 2/6 and 7) measured mainly by BLIMP-1 and AID mRNA expression. The poor survival of plasma cells *in vitro* made it difficult to analyze antibody production in cell culture supernatants and this can explain the discrepancies between Blimp-1 (**Figure 3A**) and AID (**Figure 4B**) mRNA levels and antibody production (**Figure 5A and 5B**) when B cells were treated with TLR 2/6 and TLR7 agonists.

Our results offer an explanation for the polyclonal B cell proliferation and Ig production observed during bacterial and viral infection [40]–[44]. Indeed, in 2003, Zinkernagel's group [44] showed that hypergammaglobulinemia induced during viral infection required not only a virus-induced signal but also a T cell co-stimulation, probably via CD40L. More recently, Soulas *et al.* [45], by using MyD88-deficient mice, showed that polyclonal activation of autoreactive B cells required TLR signal as well as a co-stimulation signal provided by T cells. However, our results show that the pathogen via its PAMPs not only synergizes with activated T cells to induce B cell proliferation and Ig production but also control B cell response by inducing either a B cell proliferation and activation that could induce memory B cell differentiation or production of polyclonal antibodies by bystander activation. The mechanism explaining this dissociated response is still unclear. TLR 1, 2, 4 and 6 are expressed on the cell surface, whereas TLR 3, 7 and 9 are present within the endosomal compartments. However, this difference in localization cannot fully account for differences in B cell response as in association with mCD40L, TLR2 and TLR7 agonists both induce differentiation into ASC. TLR signaling is initiated by four adapters: MyD88, TRIF (TIR domain-containing adapter inducing interferon beta), TIRAP (TIR domain-containing adapter protein) and TRAM (TRIF-related adapter molecule). MyD88 associates with all TLRs except TLR3, whereas TRIF associates with TLR3 and TLR4. TIRAP and TRAM appear to function as bridging adapters for MyD88 and TRIF, respectively. TIRAP and TRAM are essential for signaling by TLR4 and also required for TLR2 function. [46]–[47] Again, these differences in signaling adapters alone cannot explain the various B cell responses that we observe.

Finally, we showed that the addition of a BCR signal does not reorient the activation phenotype induced by TLR3 and TLR9 associated with mCD40L towards differentiation into ASC (**Figure 3B**) and even inhibits ASC differentiation induced by TLR4 *in vitro*. This effect of BCR signaling has already been described after activation of mouse B cell by TLR4 agonist associated with an anti-CD40 antibody and has been interpreted as the result of memory B cell differentiation [24]. Study of memory response *in vitro* is not possible and should be evaluated in latter *in vivo* experiments. Alternatively, BCR stimulation could favor the proliferative response at the expense of a delay in the differentiation process.

In conclusion, we show that pathogen-specific structures alone or associated with T cell co-stimulation molecules directly trigger and control B cell responses. According to the TLR pathway involved and the activation of BCR signal, the response would be directed either to memory B cells or to ASC differentiation resulting in production of mainly polyclonal antibodies. Besides a better understanding of mouse B cell biology, these observations made have important implications in vaccination by specifically designing optimally cooperative antigen/adjuvants combinations that would specifically activate B cells while limiting the polyclonal response often responsible for autoimmune diseases [48].

## Materials and Methods

### Ethics Statement

The research was conducted in accordance with the European Community guidelines (Directive 86/609/EEC) on the protection of animals used for scientific purposes. The IBMC animal house facilities are approved by French veterinary service (#E67-482-2). As no surgery or experimentation has been done on animals before euthanasia, we did not need specific ethical approval. Mice were euthanized according the European Community guidelines before spleen removing.

### Mice

BALB/c and C57BL/6 mice as well as C57BL/6 CD40 knockout mice were bred in IBMC animal house facilities.

### Antibodies and reagents

Phycoerythrin (PE)-labeled anti-mouse CD69 (clone H1-2F3), anti-mouse CD86-PE (clone GL1), anti-mouse CD138-PE (clone 281.2) and allophycocyanin (APC)-labeled anti-mouse B220 (clone RA3-6B2) monoclonal antibodies (mAbs) were purchased from PharMingen (BD Biosciences, San Diego, CA). F(ab′)2 fragment of goat anti-Mouse IgM (referred as anti-IgM in the text) used in cell culture experiments, goat anti-mouse IgG+IgM (H+L), goat anti-mouse IgM conjugated to horseradish peroxidase, goat anti-mouse IgG conjugated to horseradish peroxidase, and mouse IgM and IgG were purchased from Jackson Immunoresearch (WestGrove, PA). TLR agonists Pam_2_CSK_4_, Pam_3_CSK_4_, poly (I:C), LPS from *Escherichia coli* (K12), R848 and CpG oligonucleotide CpG2395 5′- TCGTCGTTTTCGGCGCGCGCCG- 3′ and CpG1668 5′- TCCATGACGTTCCTGATGCT- 3′ were purchased from Invivogen (San Diego, CA). Polymyxin B solution was purchased from Sigma-Aldrich (Saint-Louis, MO). Recombinant soluble hFc-mCD40L (referred as mCD40L in the text) was produced in CHO cells (Sigma-Aldrich) as previously described for Fc-EDA [49] using amino acids 115–260 of mouse CD40L.

### B cell preparation and culture

Primary B cells were purified from spleens of 10- to 12- week-old mice using a negative selection strategy. Briefly, spleen cells were depleted from macrophages, granulocytes, CD4^+^ T cells and CD8^+^ T cells by incubation during 20 minutes at 4°C with anti-CD11b (clone Mac-1), anti-GR1 (clone 8C5), anti-CD4 (clone GK 1.5) and anti-CD8 (clone Lyt-2) mAbs purified in-house. After incubation, magnetic beads coupled to anti-rat Ig (dynal, Oslo, Norway) were added during 20 minutes at 4°C. The bead-bound cells were separated by a magnet and discarded. The remaining cells referred as purified B cells (containing more than 90% B cells as determined by flow cytometry analysis) were used in all experiments. Purified B cells were cultured (3.10^5^/well) in 96-well U-bottom plates in a final volume of 200 µL or (1.15.10^6^/well) in 24-well plates in a final volume of 1150 µL. Cultures were performed in RPMI 1640 medium (Cambrex, Verviers, Belgium) supplemented with 10% fetal calf serum (FCS; Lonza, Verviers, Belgium), 10 µg/mL gentamicin (Cambrex), 10 mM HEPES (Cambrex) and 0.05 mM 2-mercaptoethanol. Cells were incubated with mCD40L alone or in association with the various TLR agonists at the concentrations indicated in the text. In some experiments, an anti-IgM antibody (1 µg/mL) was added to the culture simultaneously with TLR agonists and mCD40L. When used, anti-TLR2 antibody (1 µg/mL) or polymyxin B (20 µg/mL) were added 20 min before TLR agonists and mCD40L.

### Activation and proliferation assays

For measurement of membrane expression of activation markers by flow cytometry, cells were harvested after 24 h of culture and labeled with anti-CD69 and anti-CD86 mAb as described below. For cytokine measurement, supernatants were collected after 48 h and tested for CCL22/MDC production by ELISA. To measure cell proliferation, [^3^H]-thymidine (1 µCi; specific activity 6.7 Ci/mmol) was added after 64 h of culture, cells were harvested 8 h later on a filter with an automatic cell-harvesting device (Packard, Meriden, CT), and thymidine incorporation was assessed by using a Matrix 9600 direct beta counter (Packard; cpm range from 10 to 35,000). Data were expressed as the stimulation index calculated as follows: the mean counts per minute (cpm) of stimulated cells/the mean cpm of unstimulated cells. Proliferation experiments were performed in triplicate.

### Markers of plasma cell differentiation, CSR and SHM

To evaluate Blimp-1 mRNA expression in B cell population, purified B cells were harvested after 72 h of culture and quantitative real-time PCR (qRT-PCR) was performed as described below.

To measure IgM and IgG production by ELISA, cell culture supernatants of purified B cells cultured in 24-well plates were harvested at day 6.

In some experiments, enrichment in CD138^+^ cells was performed. For this, purified B cells were stained after 72 h of culture with PE-labeled anti-CD138 antibodies for 15 min at 4°C and further incubated with magnetic microbeads coupled to anti-PE antibodies, as indicated by the manufacturer (Miltenyi Biotech, Bergisch, Gladbach, Germany). CD138^+^-labeled cells were then selected upon cell loading on a MACS LS column (Miltenyi Biotech). Enriched CD138^+^ cells were labeled with anti-CD138 and anti-B220 antibodies before flow cytometry analysis and qRT-PCR was performed to quantify Blimp-1, Pax-5 and AID mRNAs.

### Flow cytometry

Cells were washed in phosphate-buffered saline (PBS) containing 2% FCS and incubated at 4°C for 20 min with the various antibodies used at a concentration recommended by the manufacturer. After two washes in PBS-2% FCS, cells were analyzed by flow cytometry with a FACSCalibur® and data were processed with the CellQuest 3.3 software (Becton Dickinson, Pont de Claix, France).

### ELISA assays

Commercially available ELISA reagents were employed for the determination of CCL22/MDC (Quantikine®, R&D Systems, Minneapolis, MN). All procedures were performed following the manufacturer's instructions. Results were expressed as chemokine concentration in pg/mL. The detection limit was 1.2 pg/mL.

To measure IgM and IgG in culture supernatants, 96-well plates were coated with 1 µg/mL goat anti-mouse IgG+IgM (H+L) antibody overnight at 37°C. The plates were washed and then blocked with 0.9% BSA in PBS containing 0.05% (v/v) Tween-20 for 60 min at 37°C. Supernatants were diluted in RPMI 1640 medium and added to the plates along with purified mouse IgM or IgG to establish a standard concentration curve. The plates were incubated for 60 min at 37°C, then washed, and goat anti-mouse IgM or IgG conjugated to horseradish peroxidase diluted 1/20,000 and 1/10,000 respectively in PBS-Tween, were added and incubated for 30 min at 37°C. The plates were then washed and the reaction was revealed by addition of 0.04% (v/v) H_2_O_2_ and 3, 3′, 5, 5′-tetramethyl benzidine (3 mg/mL) as chromogen. Absorbance was measured at 450 nm.

### Quantitative real-time PCR

Approximately 3.10^6^ purified B cells or 1.10^6^ enriched CD138^+^ cells were homogenized with 1 ml of Tri Reagent (Sigma-Aldrich) and total RNA was purified using RNeasy® Mini Kit (Qiagen, Germantown, MD). cDNA was synthetized by extension of a mix of oligo(dT) and random primers with ImProm-II™ reverse transcriptase (Promega, Fitchburg, WI). The reaction mix was diluted 1/10 for real time-PCR. Expression of individual transcripts was normalized according to the mean of the expression of three different housekeeping genes, namely β-actin, glyceraldehyde 3-phosphate dehydrogenase (GAPDH) and hypoxanthine guanine phosphoribosyl transferase (HPRT). Primer sequences were designed with the Primers^3^ software (http://packages.debian.org/org/fr/sid/primer3). All amplification reactions were performed in a total volume of 25 µL using a MxPro-Mx3005P Thermocycler sequence detector (Stratagene, La Jolla, CA) with Mesa Green qPCR MasterMix Plus for SYBR Assay Low ROX (Eurogentec, Seraing, Belgium) according to the manufacter's instructions. Data were analysed using the software toll rest-384-beta-9august2006.

The primer sequences (forward/reverse) used were: mBlimp-1, 5′-TTTTACTCAGCTCGCCCACCT-3′/3′-TTGGCAGGGCACACCTTACA-5′, mPax-5, 5′-GCCCACAGTCCTACCCTATTG-3′/3′-GAGGGTGGCTGTAGGGACTT-5′ mAID, 5′-GGGAGTCAAGAAAGTCACGC-3′/3′-CTGCCGTACTCTGGATGGAG-5′, mGAPDH, 5′-TGACGTGCCGCCTGGAGAAA-3′/3′-AGTGTAGCCCAAGATGCCCTTCAG-5′, mβ-actin, 5′-ATGAGCTGCCTGACGGCCAGGTCATC -3′/3′-TGGTACCACCAGACAGCACTGTGTTG-5′, mHPR, 5′-CTTGCTGGTGAAAAGGACCTCT-3′/3′-AAGTACTCATTATAGTCAAGGGCAT-5.

### Statistical analysis

Proliferation assay, activation markers expression, CCL22, IgG and IgG production in the presence or not of mCD40L were analyzed by two-way ANOVA with Bonferroni's post test using GraphPad Prism version 5.00 for Windows, GraphPad Software, San Diego California USA, www.graphpad.com. For quantitative real-time PCR analysis, the investigated transcripts are tested for significance by a pair wise fixed reallocation randomisation test ©. P<0.001 ***, P<0.01 **, P<0.05 *, P>0.05 ns.

## Supporting Information

Figure S1 **Proliferative response of mouse spleen B cells induced by TLR agonists.** (A) Purified B cells from C57/BL6 CD40 deficient or wt mice were cultured in the presence of TLR1/2 agonist Pam3CSK4 (250 ng/mL), TLR2/6 agonist Pam2CSK4 (50 ng/mL), TLR3 agonist poly (I:C) (10 µg/mL), TLR4 agonist LPS (10 µg/mL), TLR7 agonist R848 (1 µg/mL) or TLR9 agonist ODN 2395 (200 nM) alone or in the presence of 0.6 µg/mL mCD40L. (B) Purified B cells from BALB/c wt mice were cultured in the presence of TLR3 agonist poly (I:C) (50 µg/mL) and TLR4 agonist LPS (10 µg/mL) alone or in the presence of 0.6 µg/mL mCD40L, with or without Polymixin (20 µg/mL). Results are representative of at least two independent experiments.
(TIF)

Figure S2 **Mouse spleen B cell expression of BLIMP-1 mRNA.** (A) Total RNA was isolated from purified B cells from C57/BL6 CD40 deficient or wt mice cultured for 72 h with TLR1/2 agonist Pam3CSK4 (250 ng/mL), TLR2/6 agonist Pam2CSK4 (50 ng/mL) and TLR7 agonist R848 (1 µg/mL) alone or in the presence of 0.6 µg/mL mCD40L. (B) Total RNA was isolated from purified B cells from BALB/c wt mice cultured for 72 h with TLR1/2 agonist Pam3CSK4 (250 ng/mL) and TLR2/6 agonist Pam2CSK4 (50 ng/mL) alone or in the presence of 0.6 µg/mL mCD40L, with or without antagonistic anti-TLR2 antibody. (C) Total RNA was isolated from purified B cells from BALB/c wt mice cultured for 72 h with TLR4 agonist LPS (10 µg/mL) alone or in the presence of 0.6 µg/mL mCD40L, with or without Polymixin (20 µg/mL). (D) Total RNA was isolated from purified B cells from BALB/c wt mice cultured for 72 h with TLR9 agonist type B (ODN1668) alone or in the presence of 0.6 µg/mL mCD40L. Results are representative of at least two independent experiments.
(TIF)

Figure S3 **CD138 enriched mouse spleen B cell expression of BLIMP-1 and PAX-5 mRNA.** Purified B cells were cultured with TLR2/6 agonist Pam2CSK4 (50 ng/mL), TLR1/2 agonist Pam3CSK4 (250 ng/mL), TLR3 agonist poly (I:C) (10 µg/mL), TLR4 agonist LPS (10 µg/mL), TLR7 agonist R848 (1 µg/mL) or TLR9 agonist ODN 2395 (200 nM) alone or in the presence of 0.6 µg/mL mCD40L. After 72 h, population was enriched in CD138 positive cells as described in Material and Methods. Total RNA was isolated and expression of BLIMP-1 (A) or PAX-5 (B) transcripts were evaluated by quantitative real-time PCR. Results are expressed as the fold induction of gene transcription as compared to the CD138-enriched purified B cells cultured in medium. *p<0.05, **p<0.01, ***p<0,001 Results are representative of at least three independent experiments.
(TIF)

Figure S4 **Differentiation on mouse B spleen cells in ASC.** Purified B cells were cultured with TLR2/6 agonist Pam2CSK4 (50 ng/mL), TLR1/2 agonist Pam3CSK4 (250 ng/mL), TLR3 agonist poly (I:C) (10 µg/mL), TLR4 agonist LPS (10 µg/mL), TLR7 agonist R848 (1 µg/mL) or TLR9 agonist ODN 2395 (200 nM) alone or in the presence of 0.6 µg/mL mCD40L. After 72 h, population was enriched in CD138 positive cells as described in Material and Methods. CD138-enriched purified B cells were then co-stained with anti-B220 and anti-CD138 antibodies and the expression of these markers was determined by flow cytometry.
(TIF)
