# Supplementary figures and images for: Toll-Like Receptor Agonists Synergize with CD40L to Induce Either Proliferation or Plasma Cell Differentiation of Mouse B Cells
Source: PLoS One. 2011 Oct 3;6(10):e25542. doi: 10.1371/journal.pone.0025542 (PMC3184999; doi:10.1371/journal.pone.0025542)

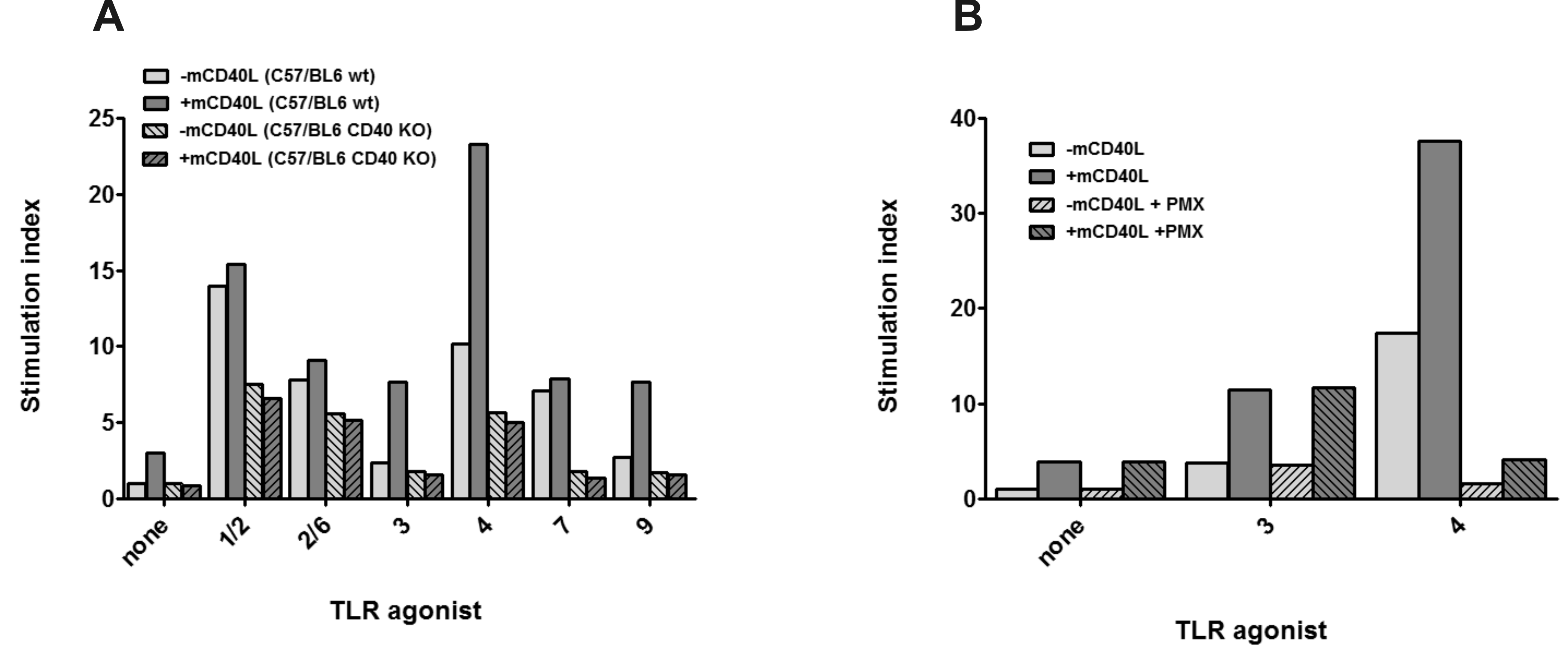

Supplement: Figure S1 — Proliferative response of mouse spleen B cells induced by TLR agonists. (A) Purified B cells from C57/BL6 CD40 deficient or wt mice were cultured in the presence of TLR1/2 agonist Pam3CSK4 (250 ng/mL), TLR2/6 agonist Pam2CSK4 (50 ng/mL), TLR3 agonist poly (I:C) (10 µg/mL), TLR4 agonist LPS (10 µg/mL), TLR7 agonist R848 (1 µg/mL) or TLR9 agonist ODN 2395 (200 nM) alone or in the presence of 0.6 µg/mL mCD40L. (B) Purified B cells from BALB/c wt mice were cultured in the presence of TLR3 agonist poly (I:C) (50 µg/mL) and TLR4 agonist LPS (10 µg/mL) alone or in the presence of 0.6 µg/mL mCD40L, with or without Polymixin (20 µg/mL). Results are representative of at least two independent experiments. (TIF) [file pone.0025542.s001.tif]

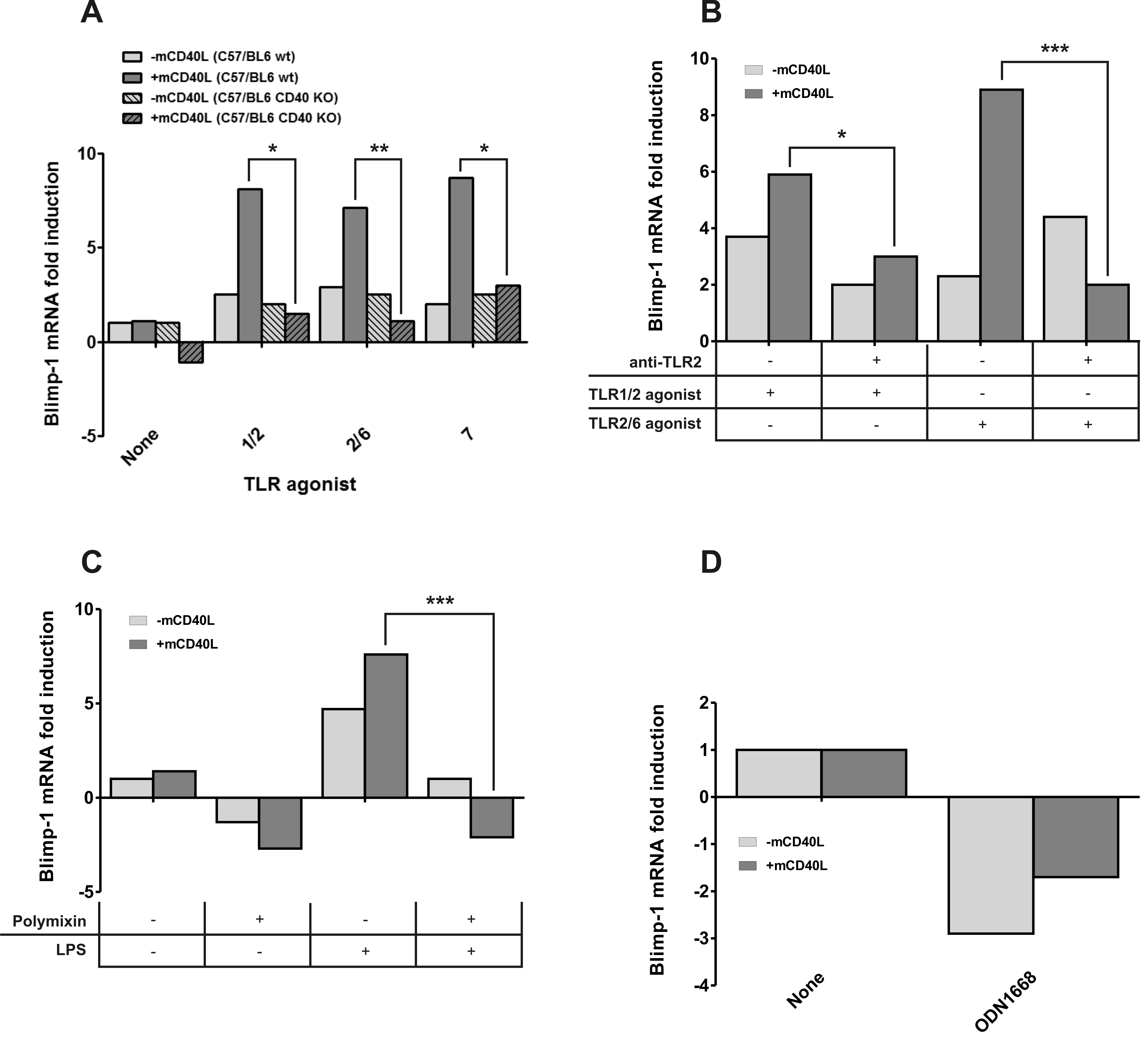

Supplement: Figure S2 — Mouse spleen B cell expression of BLIMP-1 mRNA. (A) Total RNA was isolated from purified B cells from C57/BL6 CD40 deficient or wt mice cultured for 72 h with TLR1/2 agonist Pam3CSK4 (250 ng/mL), TLR2/6 agonist Pam2CSK4 (50 ng/mL) and TLR7 agonist R848 (1 µg/mL) alone or in the presence of 0.6 µg/mL mCD40L. (B) Total RNA was isolated from purified B cells from BALB/c wt mice cultured for 72 h with TLR1/2 agonist Pam3CSK4 (250 ng/mL) and TLR2/6 agonist Pam2CSK4 (50 ng/mL) alone or in the presence of 0.6 µg/mL mCD40L, with or without antagonistic anti-TLR2 antibody. (C) Total RNA was isolated from purified B cells from BALB/c wt mice cultured for 72 h with TLR4 agonist LPS (10 µg/mL) alone or in the presence of 0.6 µg/mL mCD40L, with or without Polymixin (20 µg/mL). (D) Total RNA was isolated from purified B cells from BALB/c wt mice cultured for 72 h with TLR9 agonist type B (ODN1668) alone or in the presence of 0.6 µg/mL mCD40L. Results are representative of at least two independent experiments. (TIF) [file pone.0025542.s002.tif]

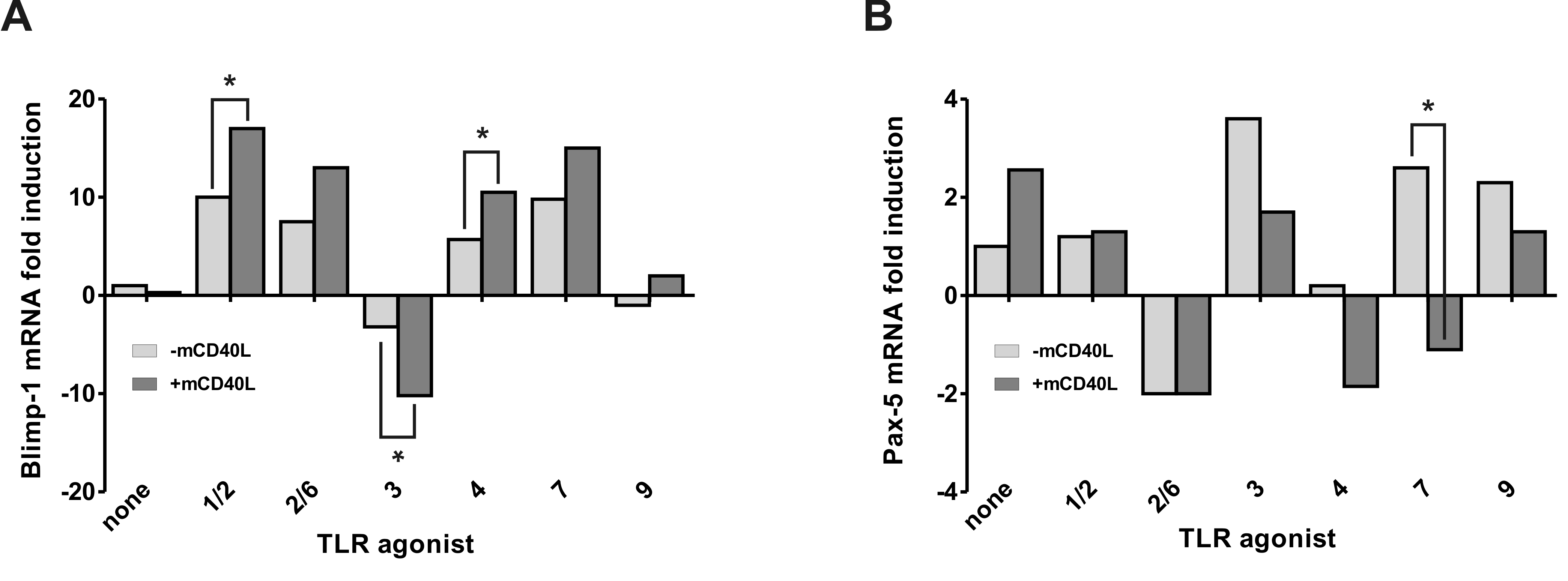

Supplement: Figure S3 — CD138 enriched mouse spleen B cell expression of BLIMP-1 and PAX-5 mRNA. Purified B cells were cultured with TLR2/6 agonist Pam2CSK4 (50 ng/mL), TLR1/2 agonist Pam3CSK4 (250 ng/mL), TLR3 agonist poly (I:C) (10 µg/mL), TLR4 agonist LPS (10 µg/mL), TLR7 agonist R848 (1 µg/mL) or TLR9 agonist ODN 2395 (200 nM) alone or in the presence of 0.6 µg/mL mCD40L. After 72 h, population was enriched in CD138 positive cells as described in Material and Methods. Total RNA was isolated and expression of BLIMP-1 (A) or PAX-5 (B) transcripts were evaluated by quantitative real-time PCR. Results are expressed as the fold induction of gene transcription as compared to the CD138-enriched purified B cells cultured in medium. *p<0.05, **p<0.01, ***p<0,001 Results are representative of at least three independent experiments. (TIF) [file pone.0025542.s003.tif]

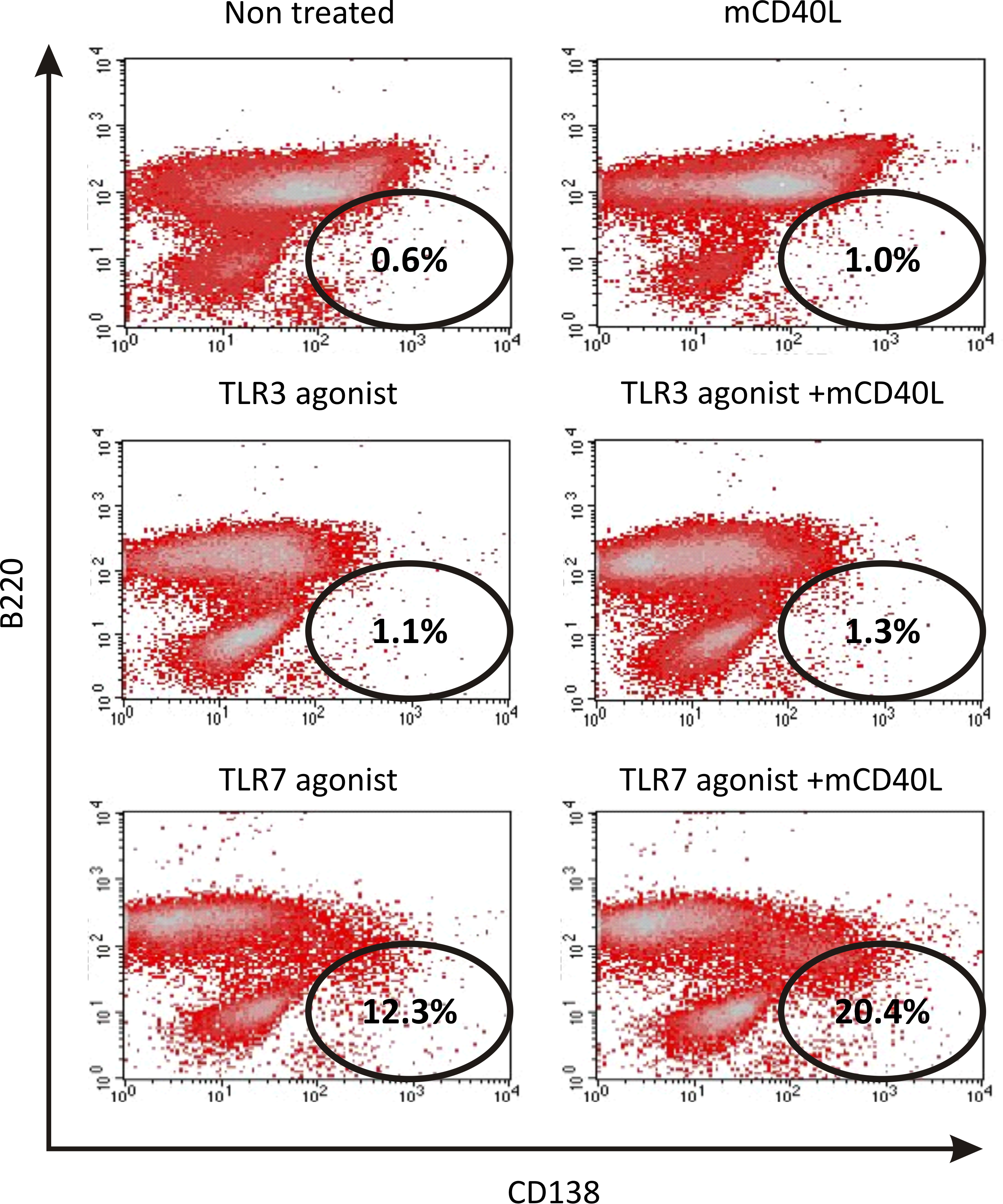

Supplement: Figure S4 — Differentiation on mouse B spleen cells in ASC. Purified B cells were cultured with TLR2/6 agonist Pam2CSK4 (50 ng/mL), TLR1/2 agonist Pam3CSK4 (250 ng/mL), TLR3 agonist poly (I:C) (10 µg/mL), TLR4 agonist LPS (10 µg/mL), TLR7 agonist R848 (1 µg/mL) or TLR9 agonist ODN 2395 (200 nM) alone or in the presence of 0.6 µg/mL mCD40L. After 72 h, population was enriched in CD138 positive cells as described in Material and Methods. CD138-enriched purified B cells were then co-stained with anti-B220 and anti-CD138 antibodies and the expression of these markers was determined by flow cytometry. (TIF) [file pone.0025542.s004.tif]
